# Wild Garlic (*Allium ursinum*) Preparations in the Design of Novel Functional Pasta

**DOI:** 10.3390/foods12244376

**Published:** 2023-12-05

**Authors:** Bojana Filipčev, Jovana Kojić, Jelena Miljanić, Olivera Šimurina, Alena Stupar, Dubravka Škrobot, Vanja Travičić, Milica Pojić

**Affiliations:** 1 Institute of Food Technology, University of Novi Sad, Blvd. Cara Lazara 1, 21000 Novi Sad, Serbia; bojana.filipcev@fins.uns.ac.rs (B.F.); jelena.krulj@fins.uns.ac.rs (J.M.); olivera.simurina@fins.uns.ac.rs (O.Š.); alena.stupar@fins.uns.ac.rs (A.S.); dubravka.skrobot@fins.uns.ac.rs (D.Š.); milica.pojic@fins.uns.ac.rs (M.P.); 2 Faculty of Technology, University of Novi Sad, Blvd. Cara Lazara 1, 21000 Novi Sad, Serbia; vanjaseregelj@tf.uns.ac.rs

**Keywords:** phytochemicals, extract, encapsulate, antioxidants, minerals, retention index, cooking behaviour, sensory, colour

## Abstract

This study investigated the design of novel pasta enriched with different forms of wild garlic (WG): a powder, an extract and an encapsulated extract applied at three enrichment levels (low/middle/high). The effect of cooking on changes in the content of bioactive compounds, antioxidative activity, cooking behaviour, texture, colour and sensory properties of the cooked pasta was evaluated. WG preparations significantly increased the antioxidant potential (by 185–600%) as well as the content of phenolics (by 26–146%), flavonoids (by 40–360%) and potassium (up to three-fold) in the cooked pasta, depending on WG type and enrichment level. Flavonoids were dominantly present in the free form. Cooking resulted in a significant loss of flavonoids (39–75%) whereas phenolics were liberated from the matrix. The highest increase in total phenolics and antioxidant activity was exerted by the WG powder and extract. Pasta hardness and adhesiveness were increased, but encapsulated WG deteriorated cooking behaviour. The best-scored enriched pasta regarding sensory quality and texture was that enriched with WG powder at the low/moderate level.

## 1. Introduction

Pasta is a popular food used in many traditional cuisines of the world and can be considered a staple food. It is available in a variety of forms, cooked as a single dish or used as a complementary ingredient in soups. It is made from wheat flour, usually durum wheat semolina, but it can be made from other cereals, with or without the addition of eggs. Moreover, pasta products are convenient food products due to their easy production, low cost and versatility of dishes. In recent years, pasta products have been innovated to deliver healthier or functional ingredients. A research work confirmed that pasta is a product particularly convenient for the incorporation of diverse functional ingredients without distinctively compromising its quality [1]. Pasta enriched with non-traditional ingredients could improve and enhance the intake of bioactive phytochemicals in an everyday diet.

Pasta enrichment has been addressed by numerous researchers with the aim of developing a health-promoting product that provides sources of antioxidants, minerals, fibres and other bioactive compounds [2]. The functional ingredients used to enrich pasta include milk and whey protein, other protein concentrates and isolates [3,4,5,6,7], legume and oilseed flours [8,9,10,11,12], dietary fibres [13,14], microalgae and seaweed [15,16,17], fruit/vegetable preparations [18,19,20,21], fish powder [22] and food industry by-products such as maize bran, defatted corn germ, corn gluten meal, grape marc, brewer’s spent grain and tomato by-products [23,24,25,26].

Vegetables have been studied as promising ingredients in the development of functional pasta. Already marketed coloured pasta (green and orange) contains spinach or carrots or tomatoes in its formulation. Studies that dealt with the enrichment of pasta with leafy vegetables included spinach and red cabbage [18], common glasswort [25], rocket [27], parsley leaves [28], dried nettle (*Urtica simensis*) leaves [29], oregano and carrot leaves [30] and dry amaranth leaves [31]. Vegetable ingredients added to pasta can have different forms: freshly chopped, dry and powdered, juice, puree, pomace, etc. The form of the ingredient affects the level of substitution in pasta formulation as well as the cooking and sensory properties of resultant pasta. Different ingredient forms are important to diversify the assortment and availability of vegetable-based ingredients for pasta manufacturers. The availability of different ingredient forms is beneficial for manufacturers in case selection has to be made depending on requirements like price, sustainability, production location, etc. Fortification with green leafy vegetables increases the content of bioaccessible phenolic compounds and contributes to the improved antioxidant capacity and biological activity of pasta. Such effects were reported in the case of pasta enrichment with 4% parsley leaves [28], an extract of common glasswort [25], 5–10% dry powdered oregano and carrot leaves [30]. In his review on current trends in the production of functional pasta, Dziki [1] praised fortification with herbs and leaves as a promising approach that results in pasta with enhanced levels of bioactive compounds and good sensory quality.

Wild garlic (*Allium ursinum*), also known as ramsons or bear’s garlic, is an edible perennial plant, which grows in forests across Europe, Asia and North America. It has green spear-shaped leaves, which can be foraged around February and March on the northern hemisphere. It has a mild taste but strong garlic odour. In culinary practise, it is used to flavour and add garlic aroma to various dishes, raw or cooked. In addition to this, wild garlic has been known in folk medicine for its health-promoting and disease-preventing properties associated with minerals (Fe, P, Na, Cu), vitamins (A, C), dietary fibres and sulphur-containing and phenolic compounds. Dominant sulphur-containing compounds are methiin and alliin, which, after cell damage and enzyme release, form a number of characteristic sulphur compounds [32]. Phenolics present in wild garlic leaves consist of free forms of ferulic and vanillic acids and bound forms of p-coumaric, ferulic and vanillic acids, and seven flavonoid glycosides were found in the n-butanol fraction of wild garlic [33]. Wild garlic exerts various therapeutic and prophylactic properties similar to those reported for garlic and onion such as an anticoagulant property due to high vitamin K content; antioxidant, anti-inflammatory and anti-aging properties due to vitamin C and phenolic compounds; laxative, depurative and antidiabetic properties due to dietary fibres, an anticancer property due to sulphur compounds; and antihypertensive, antibacterial and antibiotic properties. Few reported studies deal with biological activities of wild garlic, reviewed by Shahrarabijan [34], who demonstrate the antioxidant, cytostatic, antimicrobial and antidiabetic effects of wild garlic extracts.

This study explored pasta enrichment with various wild garlic (WG) preparations (dry powder, aqueous extract, encapsulated extract) with the aim of comparing their feasibility in pasta production and effect on pasta sensory and cooking quality. The research included an evaluation of antioxidant potential, content of total, free and bound phenolics and flavonoids and content of macro- and microelements, in addition to effects on the textural, colour and sensory properties and cooking performance of the enriched pasta. Also, attention was directed to estimating the retention of bioactive compounds, minerals and antioxidant capacity after pasta cooking.

## 2. Materials and Methods

### 2.1. Wild Garlic (WG) Preparations

#### 2.1.1. WG Powder

The leaves of wild garlic were selected, washed, rinsed and dried in a condensation drier (Optimonia, Stara Pazova, Serbia) with temperature control. For the first 2 h, drying was conducted at 30 °C and then at 45 °C until the constant mass. The total time of drying was 10 h. Drying at low temperatures contributed to the preservation of the green colour and garlic aroma. Dried leaves were milled into fine powder in a spiral jet micronising mill Disc Micronizer, Micro Powder Equipment (Ningbo Co., Ltd., Ningbo, China). WG powder was stored in air-tight glass jars protected from light at room temperature until pasta production.

#### 2.1.2. WG Extract

WG extract was prepared following the optimal extraction condition presented in research by Tomšik et al. [35]. The extraction was performed in a pressurised liquid extractor ASE 350 system Dionex Corporation (Sunnyvale, CA, USA). Briefly, 1 g of powdered wild garlic was combined with diatomic earth, following the producer’s recommendations. This mixture was then placed in 22 mL cells equipped with a stainless steel frit and a cellulose filter at the bottom to avoid the collection of suspended particles in the collection vial. Extraction was performed using acidified water (1% HCl) at a temperature of 180 °C and a pressure of 1500 psi for a duration of 10 min. The resulting wild garlic extract was subsequently stored in air-tight glass jars, shielded from light, and maintained at a temperature of −4 °C until it was used in the production of pasta.

#### 2.1.3. WG Extract Encapsulation

The encapsulated extract was prepared as described by Tomšik [36]. Prepared WG extract was encapsulated by a spray dryer (APV Anhydro AS, Søborg, Denmark) under the following conditions: atomiser speed was 20,000–21,000 rpm, inlet air temperature was set at 140 °C, while the outlet temperature was 80 °C with a constant flow of 1.36 L/h. Maltodextrin (DE 19.7) was used as a carrier at a concentration of 80% calculated on the dry residue of the extract. Encapsulated WG extract was stored in air-tight glass jars protected from light at −4 °C until pasta production.

### 2.2. Pasta Preparation

The pasta was prepared with durum wheat semolina, WG preparations and water to obtain compact, elastic and smooth dough of 29 ± 1% moisture. The control pasta was prepared identically but without the addition of WG preparations. WG preparations were applied at three levels: low, middle and high; 5–7–9% for powdered WG, 3–5–7% for encapsulated WG, and 20–30–40% for WG extract, respectively. The WG incorporation levels were defined in preliminary trials confirming that the established levels were feasible for standard pasta manufacturing conditions. The ingredients were mixed and kneaded in a pasta-making machine Pasta Fresca (Marcato, Campodarsego, Italy). The dough was sheeted into 3 mm thick dough sheets using rollers of the same machine. It was cut into strands 7 mm width and 3 mm thickness to form a tagliatelle shape. The tagliatelle samples were pre-dried for 12 min at 30 °C and then dried in a compact bakery oven with a ventilator MIWE gusto (Arnstein, Germany) at 45 °C for 240 min and the humidity level of 65%. The pasta samples of 100 g weight were packaged in sealed plastic bags and stored at room temperature.

### 2.3. Determination of Cooking Properties

Pasta cooking properties (water absorption, cooking loss, swelling index, optimum cooking time (OCT)) were determined in duplicate after cooking 100 g of pasta strands in 1 L of boiling tap water salted with 5 g of kitchen salt. The end of cooking time (optimum cooking time, OCT) was determined by observing the disappearance of the white core after squeezing between two glass plates. After cooking, pasta strands were drained, rinsed with 0.5 L of lukewarm water and left to drain for 2–3 min. The cooked pasta was weighed. Cooking and rinsing water was retained for the determination of cooking loss (CL).

Water absorption (WA) was calculated as WA (%) = (m − 100)/100, where m (g) is the mass of cooked pasta.

CL was determined by taking a 100 mL aliquot from a homogenised sample from cooking and rinsing water and pouring it into clean and weighted glass. The water was evaporated by boiling, and the glass was then dried until constant mass at 105 °C. CL was calculated according to the equation:

$$\mathrm{CL}\;(\%)=\frac{\left(m_{2}-m_{1}-K\right)\times V}{100-M}$$

where

m_1_, m_2_—mass of dry glass before and after evaporation of cooking water in grams, respectively;

K—correction for added salt, calculated as K = (4.7 × 100)/V;

V—total volume of cooking and rinsing water (mL);

M—pasta moisture (%).

Swelling index (SI) was determined as a ratio of volume of cooked and raw pasta.

### 2.4. Analysis of Phenolic Compounds

#### 2.4.1. Extraction of Phenolic Compounds

The extraction of phenolic compounds (free and bound fraction) from the pasta samples was performed according to the method described by Jambrec, Sakač, Mišan, Mandić, and Pestorić [37]. Briefly, 5 g of grounded pasta samples was mixed with 12.5 mL of EtOH:H_2_O 4:1 *v*/*v* and sonicated at 40 °C for 15 min (Elma TI-H-15, Im Garbrock, Germany). After centrifugation at 3000 rpm for 10 min (Eppendorf 5804 R, Im Steingrund, Germany), the supernatants were collected for free phenolic compounds analyses, while the solid phase was used for further extraction and the determination of bound phenolics content. The solid phase was further treated by the alkali hydrolysis protocol described by Jambrec, Sakač, Mišan, Mandić, and Pestorić [37]. The extract fractions obtained in this way were further used for the determination of the phenolic compounds and antioxidant activity of pasta.

#### 2.4.2. Determination of Total Phenolic Content

Free and bound phenolic content in pasta was determined using the Folin–Ciocalteau spectrophotometric method adapted to the microscale [38] (Tumbas Šaponjac, et al., 2016). Each well contained a mixture of 15 μL of sample, 170 μL of distilled water, 12 μL of the Folin–Ciocalteau’s reagent and 30 μL of 20% (*w*/*v*) sodium carbonate. The prepared microplate was incubated for 1 h, and the absorbances were measured at 750 nm. Distilled water was used as blank. The obtained results were expressed as gallic acid equivalents (GAE) per 100 g of pasta.

#### 2.4.3. Determination of Total Flavonoid Content

Flavonoid content in the free and bound fraction was determined using the aluminium chloride colorimetric assay adapted for a 96-well microplate: 5 mL of extract, 1 mL of distilled water and 2.5 mL of AlCl_3_ solution (26.6 mg AlCl_3_ × 6H_2_O and 80 mg CH_3_COONa dissolved in 20 mL distilled water). A blank probe was prepared by replacing the AlCl_3_ solution with distilled water. The absorbance of the probes and blank probe were measured immediately at 430 nm. Results were expressed as rutin equivalents (RE) per 100 g of pasta.

### 2.5. Determination of Antioxidant Activity

The antioxidant activity was calculated as the sum of the free and bound fraction activities.

#### 2.5.1. DPPH Radical Scavenging Assay

The DPPH radical scavenging assay was performed spectrophotometrically according to Tumbas Šaponjac et al. [38]. Briefly, 250 μL DPPH• solution in methanol (0.89 mM) was mixed with 10 μL of sample in a microplate well. Absorbance was measured at 515 nm after 50 min incubation in the dark at ambient temperature. Methanol was used as a blank. DPPH radical scavenging activity values were calculated using the following equation:

DPPH = [(A_control_ − A_sample_)/A_control_] × 100

where A_control_ is the absorbance of the blank and A_sample_ is the absorbance of the pasta sample. The results were expressed in μmol Trolox equivalent (TE) per 100 g of pasta. Analyses were performed in three replicates.

#### 2.5.2. Reducing Power

Reducing power (RP) was determined by the method of Oyaizu [39] adapted for a 96-well microplate. In brief, a 25 μL sample or 25 μL water (blank test), 25 μL sodium phosphate buffer (pH = 6.6), and 25 μL of 1% potassium iron(III) cyanide were mixed and incubated in a water bath for 20 min at 50 °C. After cooling, 25 μL of 10% trichloroacetic acid was added, and solutions were centrifuged at 2470× *g* for 10 min. After centrifugation, 50 μL of supernatant was mixed with 50 μL of distilled water and 10 μL of 0.1% iron(III) chloride in the microplate. Absorbances were measured immediately at 700 nm. The results were expressed in μmol Trolox equivalent (TE) per 100 g of pasta. Analyses were performed in three replicates.

#### 2.5.3. ABTS Radical Scavenging Assay

The ABTS radical scavenging assay was evaluated employing a modified method according to Tumbas Šaponjac et al. [40]. The absorbances of 250 μL activated ABTS^+●^ (with MnO_2_) before and 35 min (incubated at 25 °C) after the addition of 2 μL of sample were measured at 414 nm. Distilled water was used as the blank. The results were expressed as μmol Trolox equivalent (TE) per 100 g of pasta. Analyses were performed in three replicates.

### 2.6. Determination of Minerals

Minerals content (K, Ca, Mg, Fe and Zn) in the wild garlic-enriched pasta were detected and quantified by an atomic absorption spectrometer (Varian spectra AA 10, Varian Techtron Pty Limited, Melbourne, Australia). The analytical method for metal content determination was following the standard method [41]. All measurements were conducted in triplicate, and mean values were given.

### 2.7. Texture Measurements

The textural properties of cooked pasta were determined by a compression test on a texture analyser TA.XTplus (Stable Micro Systems, Godalming, UK) equipped with a 36 mm cylinder probe. The pasta was cooked at optimum cooking time before the test. Two strands of cooked pasta were compressed with the probe at 2 mm/s cross-head speed to 75% strain. From the force–time curves, a maximal force was recorded and interpreted as pasta hardness. The area in the negative region of the plot recorded during probe redraw was calculated and taken as an indicator of adhesiveness. The measurements were performed in ten replicates.

### 2.8. Colour Determination

Pasta surface colour (L*, a*, b*) was determined, before and after cooking, using a Chroma Meter Konica Minolta CR-400 (Minolta, Tokyo, Japan) with a CR-A33f attachment calibrated with a white standard plate CR-A43 D65 illumination and 10° standard observer angle. Measurements were performed in triplicate.

### 2.9. Sensory Evaluation

Sensory evaluation was approved by the ethical committee (175/I/31-3). Descriptive sensory analysis was performed with trained panellists (7 females and 1 male, at the age of 28–45) in a sensory laboratory equipped with all necessary facilities ISO 8589 [42] under artificial daylight and temperature control (22 °C). Assessors received a list of sensory descriptors which focused on different aspects of appearance, taste, odour, flavour, and texture of uncooked and cooked pasta. During open sessions, assessors discussed descriptors appropriateness, and the final list was reached (Table 1). The intensities of selected attributes were expressed by using a 10 cm line scale anchored at both ends. The sensory evaluation was conducted as a balanced factorial design within three consecutive days. The order of a sample presentation was specified by the Experiment design for sensory analysis with XLSTAT-MX (XLSTAT 2018.7). Every assessor evaluated four different pasta samples per session, and evaluation was performed in two replications. The pasta samples (20 g of each) were delivered individually on a white plastic plate (uncooked pasta) or in the thermal plastic cups (cooked pasta; served within 15 min after cooking), which were coded with three randomly chosen numbers. Room-temperature water was used for palate cleansing. All assessors received written information about the study, and they signed informed consent to participate.

### 2.10. Statistical Analysis

The data obtained were analysed using a two-way analysis of variance (ANOVA) and Tukey’s HSD test for mean comparison (*p* < 0.05) and Pearson’s coefficient correlation. All statistical analyses were performed using the statistical software Statistica 13 (TIBCO Software Inc., Palo Alto, CA, USA).

All sensory data were processed statistically using the software package XLSTAT 2018.7. One-way ANOVA and Tukey’s HSD test for a comparison of sample means were used to analyse variations among sensory profiles of the investigated pasta samples. The descriptive sensory attributes were submitted to principal component analysis (PCA) to obtain a sensory map. The level of statistical significance for all performed statistical analyses was set at 0.05.

#### Calculation of True Retention Index

The true retention index accounts for changes in sample weight during thermal treatments providing a more realistic estimation of nutrient content in the prepared food. The true retention was calculated according to the equation according to Murphy et al. [43]:

$$TRUE\;RETENTION\left(\%\right)=\frac{phenolic\;content\;per\;g\;of\;treated\;food\times g\;of\;food\;after\;treatment}{phenolic\;content\;per\;g\;of\;raw\;food\times g\;of\;food\;before\;treatment}\times 100$$

## 3. Results and Discussion

### 3.1. Bioactive Compounds and Antioxidative Activity of Pasta Enriched with WG

#### 3.1.1. Phenolic and Flavonoid Compounds

**Figure 1:**
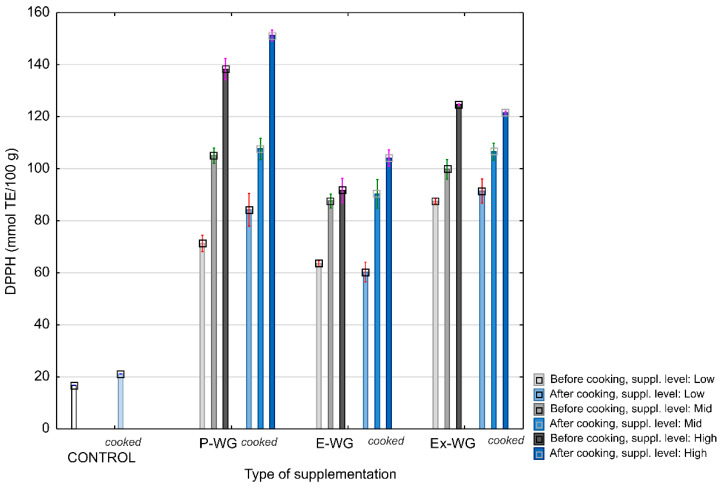
Effect of enrichment and cooking on the antioxidative activity of pasta. WG: wild garlic, P-WG: powdered WG, E-WG: encapsulated WG, Ex-WG: extract. Enrichment levels (Low–Mid–High): 5–7–9% powdered WG; 3–5–7% encapsulated WG; 20–30–40% WG extract.

The results presented in Table 2 and Figure 1 show that supplementation with WG powder, extract and encapsulate was a successful attempt to increase the phenolic and flavonoid content as well as the antioxidant potential of pasta. The total phenolic content (TPC), total flavonoid content (TFC) and antioxidant activity of the investigated samples showed enriched pasta to have a significantly higher total phenolic and flavonoid content and antioxidant potential compared to the control sample. An important improvement in TPC is observed with the increase in the percentage of supplementation in all investigated samples. As compared to the control pasta, the lowest increase in polyphenolic compounds exceeded 25% for pasta enriched with a low level of powdered WG, while the highest supplementation level in all investigated enriched pasta resulted in at least twofold higher TPC. The TPC of investigated samples was in the range 1.34 to 2.61 mg GAE/g d.m. The highest TPC was observed in the pasta enriched with the highest WG extract content (2.61 mg GAE/g d.m.), which was followed by encapsulated garlic extract samples (2.46 mg GAE/g d.m.) and then powdered WG (2.15 mg GAE/g d.m.). Analysis of TPC showed that the enriched pasta samples, especially samples with powdered wild garlic and encapsulated wild garlic extract, contained slightly more phenolic in free form. Present free phenolics are assumed to be gallic acid, *p*-coumaric and ferulic acids, which were reported as dominant in WG [44] and have been attributed to the health benefits due to their antioxidant properties [45]. The slightly increased content of bound phenolics in the enriched pasta with extracted WG (extract and encapsulate) might not be attributable to WG supplements but to structural rearrangements between the bound phenolics in the matrix.

The total flavonoids followed the same trend as the TPC, i.e., an increase in enrichment increased the TFC (Table 2). However, the samples with the highest TFC were those with the encapsulated extract (0.23 mg RU/g d.m.), which was closely followed by those with powdered wild garlic (0.22 mg RU/g d.m.). The analysis confirmed that the TFC in the investigated samples was in free forms, and around 10% or less were bound. This may be the consequence of pasta processing and cooking, which can affect the bioactive compounds composition and antioxidant properties of the final product [46]. According to research by Tomšik et al. [35] and Oszmiański, Kolniak-Ostek, and Wojdyło [47], the flavonoids in the investigated samples most probably present kaempferol derivatives.

An increase in antioxidant activity is one of the main aims of pasta supplementation. As depicted in Figure 1, increasing the supplementation level of WG supplements led to an increase in the antioxidant activity of pasta against DPPH. This increase in antioxidant activity compared to the control one was the most prominent in the samples with powdered WG (from 4 to 7-fold increase), which was followed by paste enriched with extract (from 4 to ≈6-fold increase). The antioxidant activity of enriched pasta can be associated with the content of TPC and TFC. DPPH values in the cooked pasta were highly correlated (*p* < 0.05) to TPC (r^2^ = 0.86), free phenolics (r^2^ = 0.78), bound flavonoids (r^2^ = 0.77), and moderately to TFC (r^2^ = 0.65) and free flavonoids (r^2^ = 0.63), suggesting that free phenolics and bound flavonoids were the primary contributors to DPPH antiradical activity. The high increase in the antioxidant capacity of the enriched pasta can be linked to the presence of phenolic compounds with prominent antioxidant activity such as certain flavonoids; however, a moderate correlation between antioxidant activity and TF suggests that the sulphur compound, which among other compounds originated from wild garlic, likely plays a role in the antioxidant properties of the supplemented pasta [35].

A high increase in the TPC (67%) and antioxidant activity (146% in ABTS assay) was also noted by Sęczyk and co-workers [28] after the enrichment of wheat pasta with 4% dried parsley leaves. The incorporation of extract of common glasswort (*Salicornia europea*) into durum wheat pasta led to a 1.2-fold increase in TPC, a 2.4-fold increase in TFC and almost a 2.5-fold increase in the antioxidant activity (FRAP assay) after in vitro digestion.

#### 3.1.2. Retention of Bioactive Compounds and DPPH upon Pasta Cooking

**Figure 2:**
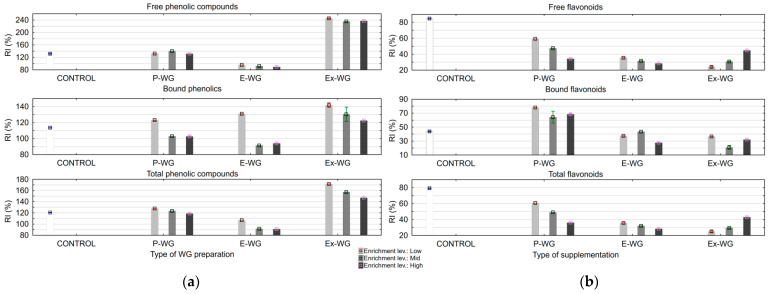
Effect of cooking on the retention of flavonoid (**a**) and phenolic (**b**) compounds in enriched pasta (RI—true retention index, P—powdered WG, E—encapsulated WG, Ex—WG extract. Enrichment levels—(Low–Mid–High): 5–7–9% powdered WG; 3–5–7% encapsulated WG; 20–30–40% WG extract).

To obtain insight into the preservation of bioactive compounds during cooking, their true retention index (TRI) was determined in the samples of WG-enriched pasta. The TRI was calculated taking into account the changes in pasta weight during cooking [43], which are the consequence of solid loss and moisture gain in the case of pasta cooking. The results showed that generally, the TRI values for free and bound forms of phenolics were over 100% (Figure 2). The TRI for phenolics was the highest in pasta enriched with extract (above 200% for free phenolics), which was followed by pasta with powdered WG. There was higher retention for free phenolics. The possible explanation may be the increased extractability and/or release of compounds from bound forms as a consequence of boiling. In pasta with encapsulated WG at moderate and high supplementation levels, the TRI values were below 100% (from 88.7% for free phenolics to 93.9% for bound phenolics). Nevertheless, high TRI (130%) values for bound phenolics were observed in pasta with a low supplementation level of encapsulated wild garlic. The lower retention of phenolic compounds for encapsulated WG extract might be due to the use of maltodextrin as a wall material for encapsulation. The use of maltodextrin alone resulted in less effective encapsulation and a low retention of reactive and volatile compounds [19,48,49].

In contrast to the TPC, the concentration of TFC decreased as a consequence of pasta cooking, which was probably due to leaching or thermal degradation. The highest retention of flavonoids in all forms was observed in pasta with powdered WG: 78.1% for bonded flavonoids and around 60% for free and total flavonoids at the lowest supplementation level. The TRI declined with higher supplementation levels of powdered garlic. In contrast, the TRI for all forms of flavonoids increased in pasta with extracts as supplementation levels increased.

Roccheti et al. [50] examined the fate of bound and free phenolics in gluten-free pasta from different ingredients upon cooking and concluded that the differences in the phenolic profile were more affected by the food matrix than the bound or free form of phenolic compounds. They mainly recorded a reduction in all phenolic compounds as the consequence of boiling but at different magnitudes depending on the matrix. In the majority of literature data, a decrease in total phenolics in pasta after cooking was reported [51,52,53,54]. Similarly to the results of this study, Khan et al. [50] observed an increase in bound phenolics in pasta upon cooking and speculated that boiling can enhance the extractability of bound phenolics from the food matrix. Prabhasankhar [52] concluded that the addition of wakame powder to pasta contributed to the retention of phenolic compounds in pasta after cooking.

In the WG-enriched pasta from our study, the antioxidant capacity mostly increased after cooking except in the case of pasta with encapsulated extract at a low supplementation level. Cooking has probably induced a release of bound phenols and therefore resulted in increased antioxidant activity. An increase in antioxidant capacity after cooking was also reported by Bustos et al. [55].

### 3.2. Mineral Profile of Pasta Enriched with Wild Garlic

#### 3.2.1. Mineral Profile

Green leafy vegetables are known as a good source of minerals and vitamins and when consumed regularly, they can substantially improve the micronutrient status of the population [17]. Supplementation with dry herbs or their extracts can improve the nutritional value of pasta, which was confirmed in the present research.

The mineral profile of enriched pasta after cooking is shown in Table 3. The results indicate that even after thermal processing, the mineral composition of enriched pasta samples remained high. The results indicated that K, Mg and Ca were the most represented macroelements in the WG-enriched pasta, and a marked increase (*p* < 0.05) was observed compared to the control sample. The mineral contents of produced pasta samples were significantly affected (*p* < 0.05) by supplementation level, and rising levels of added WG supplements significantly increased the amount of K, Mg, Ca, Fe and Zn. Similar findings related to the content of Ca, Fe and Zn were reported in the case of pasta supplementation with dried leaves of stinging nettle at 5–20% levels [29].

Looking more closely at the mineral composition of cooked pasta samples, the highest increase was observed in K content, and it was most prominent in the samples enriched with powdered WG. In pasta enriched with powdered WG, the K content increased more than twofold at the lowest supplementation level, while at the highest supplementation level; the potassium content increased threefold compared to the control sample. The WG extract and encapsulate doubled potassium at the highest supplementation level, while low and middle supplementation exerted a moderate but significant increase in K. Comparing the efficiencies of WG extract and encapsulated WG extract, it was observed that these supplements provided similar enrichment in K at the same supplementation level.

Calcium and magnesium content followed the trend of the potassium content increase. The increase was observed in pasta samples with the highest enrichment levels. In comparison to the control, powdered WG contributed to Ca increase by 13–20%, i.e., 16–24% for Mg, across applied supplementation levels.

The results revealed that the Fe content increased up to double in pasta with WG powder, while less prominent increases were observed in pasta enriched with extract of WG. The highest increase in Zn was achieved by encapsulated garlic, which was followed by powdered garlic.

#### 3.2.2. Retention of Minerals upon Pasta Cooking

The thermal treatment of pasta by boiling in water and subsequent rinsing and discarding of cooking water has been suspected as a cause of significant nutrient losses. Early studies by Ranhotra et al. [56,57] reported on the significant loss of potassium upon pasta cooking, ranging from 50% to as high as 70%. Our study confirmed these findings, since the true retention of K ranged from 24.5% to 48.7% (Table 3). Much higher retentions (above 80%) were observed for the other studied minerals (Ca, Mg, Fe and Zn), similar to the findings of Ranhotra et al. [57]. According to Yaseen [58], the typical retention of minerals in macaroni was around 70% except for K and Na. Here, in the case of Ca, Mg and Zn, occasionally TRI values above 100% were calculated, which can be a consequence of mineral uptake from tap water. Hard tap water was recognised as a possible cause of high mineral retention in prepared meals [59].

### 3.3. Cooking Properties of WG-Enriched Pasta

The inclusion of different preparations of WG changed the cooking behaviour of enriched pasta. Table 4 presents the attributes that describe the cooking quality of enriched pasta. Optimum cooking time (OCT) reduced from 13 min (control) to 5–12 min. The most affected were samples with added wild garlic powder followed by those with extract. A decrease in OCT has been frequently observed in enriched pasta, especially that with increased dietary fibre content due to disruption of the gluten network and consequent easier water penetration causing early starch gelatinisation [60]. The fibre content in wild garlic powder probably caused a prominent decrease in optimum cooking time.

Cooking loss (CL) is an important attribute in pasta quality assessment. According to Dick and Youngs [61], an acceptable limit for cooking loss in pasta is up to 8%. CL in wild garlic-enriched pasta ranged between 6 and 9.7% (Table 4). In comparison to the control, CL was significantly higher in the enriched pasta with the exception of pasta with extract at moderate and high enrichment levels. However, only pasta with encapsulated WG at moderate and high supplementation levels exceeded the 8% limit. Cooking loss depends on the ability of the gluten network to entrap starch granules and polyphenols and thus limit their leaching to cooking water [62]. Non-starchy and fibre-rich ingredients increase cooking loss due to the disruption of the gluten network and its lower ability to retain starch granules [55,60]. High-antioxidant ingredients like oregano and carrot leaf decreased cooking time and increased cooking loss in enriched pasta [30]. In our study, significant correlations were found between CL and free phenolic acids (r^2^ = 0.43, *p* < 0.05) i.e., free/total flavonoids (r^2^ = 0.67/0.69, *p* < 0.05, respectively).

The swelling index (SI) of pasta is an indicator of water absorbed by the starch and proteins during cooking, which is utilised for two competitive and antagonistic transformations: starch gelatinisation and protein hydration. The pasta cooking quality is determined by the prevalence of one of these mechanisms: if the hydration and coagulation of proteins are dominant, a protein matrix will be established which entraps starch granules and allows their gradual gelatinisation, manifesting good-quality pasta. If, however, the gluten matrix tends to weaken, starch gelatinisation and solubilisation prevail, then amylose leaches to the cooking water while amylopectin remains on the surface, causing adhesiveness and resulting in low-quality, sticky, watery pasta [55]. SI is closely related to water absorption (WA). SI and WA were reduced in pasta with powdered and encapsulated wild garlic in comparison to the control. In contrast, pasta with extract showed higher WA and SI. Bianchi et al. [60] related the increased SI in pasta with increased water absorption and faster gelatinisation of starch granules due to a weakened gluten network. Indeed, polyphenols, as strong reducing agents, have been shown to diminish gluten strength and stability by reducing disulphide cross-links and interrupting non-covalent interactions [63]. However, the low values for cooking loss for pasta with garlic extract, especially at higher enrichment levels, did not support the hypothesis of a disrupted gluten network. It could be speculated that polyphenols in WG extract contributed to the reinforcement of gluten network and decreased the cooking loss. Liu et al. [64] reported that although matcha tea powder generally weakened the structure of gluten due to reduction action, isolated specific active components in matcha like L-theanine and polyphenols improved the elasticity and toughness of gluten by promoting the lamellar and grid structure of gluten by intermolecular hydrogen bonds. SI and WA were significantly negatively correlated to total, bound and free flavonoids in enriched pasta (r^2^ = −0.57/−0.67 i.e., −0.67/−0.72, *p* < 0.05). According to Snelders et al. [65], phenolic acids exert an impact on gluten functionality regardless of their extractability status (free or bound phenolics).

Pasta hardness was altered by the WG preparations used (Table 4). In comparison to the control, a significant increase was reached in the case of enrichment with moderate and high levels of WG powder and high levels of extract. In contrast, lower and middle levels of encapsulated garlic decreased pasta hardness, which may be due to the presence of maltodextrin as a carrier in the encapsulate matrix. Adhesiveness increased upon the addition of WG preparations: a significant increase was observed in the pasta enriched with powdered WG at all levels as well as for high supplementation levels of encapsulated and extracted WG. Actually, at high supplementation levels, all investigated WG preparations significantly increased pasta hardness and adhesiveness compared to the control, and there was no significant difference in the texture within the enriched pasta. Han and co-workers [66] reported an increase in all the observed textural properties (hardness, adhesiveness and chewiness) in noodles enriched with fine green tea powder, green tea solubles and extracted tea polyphenols. They noted that tea polyphenols exhibited the most prominent influence on pasta textural features because polyphenols were able to enhance the microstructure of the gluten network by promoting hydrophobic and hydrogen bonds interactions and water–solid interaction [66]. On the other hand, Xu et al. [67] observed a decrease in Chinese white salted noodle hardness and no effect on adhesiveness upon the addition of different levels (0.5–2%) of tea extracts (green tea, black tea, oolong tea). They speculated that noodle softening might have been caused by higher starch pasting viscosity and a reducing action of tea polyphenols that disintegrated the SS bonds and diminished gluten strength. In our study, pasta hardness was positively correlated to the total phenolics and flavonoids and their different forms (free, bound) except for bound phenolics. Adhesiveness was significantly positively correlated with phenolics and flavonoids regardless of their form. The highest correlations were observed between adhesiveness and bound flavonoids and DPPH (r^2^ = 0.79 and 0.75, *p* < 0.05, respectively).

### 3.4. Colour of Pasta Enriched with WG

The colour characteristics of non-cooked and cooked pasta are displayed in Table 5. Colour is a very important attribute of pasta quality that impacts appearance and overall acceptability. The colour intensity is defined by the intensity of red (a*), yellow (b*) hue and lightness (L*). The L* values of both uncooked and cooked pasta were around 72. According to Kolarič [68], L* values above 60 are desirable. The various studied WG supplements significantly decreased L* except in the case of extract at a low enrichment level in cooked pasta. Powdered and encapsulated WG supplements decreased L* below 55 in both cooked and non-cooked pasta, which indicates a rather dark pasta according to Charles et al. [69]. Increased darkness is common after pasta cooking because of the formation of Maillard reaction products. Redness was differently affected by supplementation with various WG preparations: encapsulate and extract significantly increased a*, whereas powdered WG decreased the a* value. Cooking reduced redness (a*) and yellowness (b*) in control and enriched pasta, which is probably due to the leaching and thermal degradation of pigments [70]. The change in a* upon cooking was the lowest in the case of powdered WG. The highest yellow colour (over 34) was exhibited by pasta with WG extract before cooking, which was followed by the non-cooked control. Pasta with powdered WG was the darkest in colour, and least colourful, with the lowest yellowness and more greenish nuances probably due to the high concentration of natural pigments present in the WG powder.

### 3.5. Sensory Analysis

**Figure 3:**
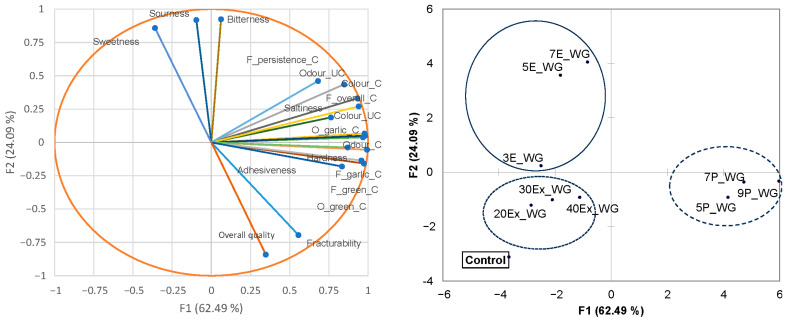
Variable (**left**) and sample (**right**) plots of the first two principal components extracted by applying principal component analysis on sensory descriptive data for pasta enriched with different WG preparations. On the sample plot: circle with full line (-) denotes pasta with encapsulated WG; circles with dotted lines (---) pasta with WG extract and (···) pasta with powdered WG.

The impact of the form in which WG was used in pasta formulation (extract, encapsulate or powder) and enrichment level on pasta appearance, taste, odour, flavour and texture was assessed by the sensory panel. The obtained descriptive data (Figure 3) indicate that the sensory panel was able to discriminate pasta samples based on their sensory characteristics. Samples were mostly separated based on their odour, flavour and taste characteristics. Pasta samples containing powdered WG at all enrichment levels (5P_WG, 7P_WG and 9P_WG, right side on the map) were associated with the green colour, intense garlic and green flavour and odour, and highly pronounced overall odour especially perceived after cooking. Contrary to this, samples containing extracts of WG at all enrichment levels (20Ex_WG, 30Ex_WG, and 40Ex_WG) sit in the middle of the sensory map, were characterised by a yellowish colour, and were associated with moderate odour and flavour attributes. Samples containing encapsulated WG were characterised by a yellowish–brown colour and possessed distinct bitterness, especially at the middle and high enrichment levels (5E_WG and 7E_WG), and a more noticeable sweet and sour taste in comparison to the other pasta samples. Regarding overall quality, the control sample showed significantly (*p* < 0.05) the highest overall quality, which was followed by WG powder and the extract-containing samples. The quality-lowering factors were primarily intensity of bitterness (r = −0.705), sourness (r = −0.779) and sweetness (r = −0.916), which resulted in the poor overall quality of pasta enriched with encapsulated WG. The low sensory performance of pasta with encapsulated WG extract was surprising since the encapsulated WG came from maltodextrin, which is known for its mild and neutral taste. This outcome might be associated with the existence of a specific taste–taste interaction between maltodextrin and WG aroma compounds. While maltodextrin suppressed to some extent the wild garlic odour and taste and conferred more sweetness, it might have been less effective in masking bitter and sour off-flavours, resulting in an astringent note. It was reported that maltodextrin was less effective in masking the strong odour and taste of microencapsulated garlic in comparison to whey protein isolate and whole milk protein [71].

## 4. Conclusions

This study has demonstrated that enrichment with wild garlic may provide higher functional potential to pasta by increasing the content of phenolics, flavonoids and minerals as well as by improving its antioxidant activity. Thermal treatment by cooking affected the preservation of these compounds; phenolic compounds and antioxidative action against DPPH were increased upon cooking, while flavonoids and some minerals were partly lost. The cooking behaviour was affected by the type of WG preparations and enrichment level. A lower swelling index and water absorption, increased cooking loss and shorter cooking time were mainly characteristic of the enriched pasta. The highest cooking loss (above 8% acceptance limit) was exerted by the pasta with encapsulated WG when applied at the middle and high doses, while the pasta with the WG extract had a similar swelling index and water absorption as the control pasta. The powdered WG and extract changed the colour of the pasta, giving lower brightness and green tones, whereas encapsulated WG resulted in brighter pasta with the highest redness without green nuances. WG preparations affected the pasta texture by increasing their hardness and adhesiveness except in pasta with encapsulated WG at low and middle enrichment levels, which were softer than the control and similarly adhesive. Sensory analysis showed that the best-scored enriched pasta was prepared with powdered WG, which was followed by WG extract. The encapsulated WG gave a bitter taste to pasta, which resulted in a reduced overall quality.

Summarising the results, it could be concluded that the powder and aqueous extract are the most preferable forms of WG preparations in the design of functional pasta; they are able to provide pasta with good cooking properties, sensory acceptance and a high content of bioactive compounds as well as retention after cooking.

## Figures and Tables

**Table 1:** Sensory attributes definitions and end anchors used in sensory analysis of pasta samples.

Sensory Attributes	Descriptors with Abbreviation	Definition with End Anchors
Appearance	Colour nuance (uncooked pasta) (Colour_UC)	The nuance of colour from yellow to green.
	Colour nuance (cooked pasta) (Colour_C)	
Odour	Overall odour intensity of uncooked pasta (Odour_UC)	The overall intensity of odour associated with cereals and added raw material. (*none–intensive*)
	Overall odour intensity of cooked pasta (Odour_C)	The overall intensity of odour associated with cereals and added raw material topped with boiling water. (*none–intensive*)
	Garlic odour intensity (O_garlic_C)	The intensity of odour associated with fresh wild garlic. (*none–intensive*)
	Green odour intensity (O_green_C)	The intensity of odour associated with green sprouts or something unripe. (*none–intensive*)
Taste	Bitterness	The intensity of bitter taste associated with caffeine solution. (*none–intensive*)
	Saltiness	The intensity of savoury taste associated with sodium chloride solution. (*none–intensive*)
	Sweetness	The intensity of sweet taste associated with sucrose solution. (*none–intensive*)
	Sourness	The intensity of sour taste associated with citric acid solution. (*none–intensive*)
Flavour	Overall flavour intensity (F_overall_C)	Overall intensity of flavour associated with cereals and added raw material topped with boiling water. (*none–intensive*)
	Garlic flavour intensity (F_garlic_C)	The intensity of flavour associated with wild garlic assessed during mastication. (*none–intensive*)
	Green flavour intensity (F_green_C)	The intensity of flavour associated with green sprouts or unripe fruit assessed during mastication. (*none–intensive*)
	Flavour persistence (F_persistence_C)	The persistence of flavour perceived after pasta swallowing measured in seconds. (*short–long*)
Texture	Fracturability (uncooked pasta)	Capability of being bent and returning to original structure of the pasta strands. (*not at all–very*)
	Hardness	Force required biting down on pasta strands between the molars. (*not at all firm–very firm*)
	Adhesiveness	Degree to which pasta strands adhering to the molars during mastication. (*not at all adhesive–very adhesive*)
Quality	Overall quality	The overall assessment that takes into consideration all the components perceived. (*low–high*)

**Table 2:** Phenolic and flavonoid compounds in cooked pasta enriched with wild garlic (WG) preparations.

	Pasta Types									
	Control	Powdered WG			Encapsulated WG Extract			WG Extract		
		Low	Mid	High	Low	Mid	High	Low	Mid	High
Phenolic compounds (mg GAE/g d.m.)										
Total	1.06 ± 0.001 ^a^	1.34 ± 0.006 ^b^	2.04 ± 0.009 ^e^	2.15 ± 0.007 ^f^	1.70 ± 0.006 ^c^	2.21 ± 0.002 ^g^	2.46 ± 0.010 ^h^	1.86 ± 0.012 ^d^	2.22 ± 0.002 ^g^	2.61 ± 0.012 ^i^
Free	0.44 ± 0.005 ^a^	0.74 ± 0.005 ^b^	1.28 ± 0.007 ^g^	1.33 ± 0.003 ^h^	1.03 ± 0.004 ^f^	1.33 ± 0.000 ^h^	1.40 ± 0.005 ^i^	0.76 ± 0.007 ^c^	0.85 ± 0.002 ^d^	0.92 ± 0.003 ^e^
Bound	0.62 ± 0.005 ^a^	0.60 ± 0.001 ^a^	0.76 ± 0.001 ^b^	0.82 ± 0.004 ^b, c^	0.67 ± 0.002 ^a^	0.88 ± 0.002 ^c^	1.06 ± 0.005 ^d^	1.10 ± 0.028 ^d^	1.38 ± 0.099 ^e^	1.71 ± 0.010 ^f^
Flavonoids (mg RU/g d.m.)										
Total	0.05 ± 0.000 ^b^	0.12 ± 0.001 ^f^	0.18 ± 0.001 ^g^	0.22 ± 0.004 ^f^	0.08 ± 0.000 ^d^	0.20 ± 0.002 ^h^	0.23 ± 0.003 ^i^	0.04 ± 0.002 ^a^	0.07 ± 0.000 ^c^	0.10 ± 0.000 ^e^
Free	0.05 ± 0.000 ^b^	0.11 ± 0.000 ^f^	0.17 ± 0.000 ^g^	0.20 ± 0.001 ^i^	0.08 ± 0.000 ^d^	0.19 ± 0.002 ^h^	0.22 ± 0.003 ^j^	0.04 ± 0.002 ^a^	0.06 ± 0.000 ^c^	0.09 ± 0.000 ^e^
Bound	0.004 ± 0.000 ^a^	0.012 ± 0.000 ^d,e^	0.016 ± 0.001 ^e^	0.021 ± 0.000 ^f^	0.007 ± 0.000 ^a–c^	0.012 ± 0.000 ^c–e^	0.012 ± 0.000 ^d,e^	0.005 ± 0.000 ^a,b^	0.006 ± 0.021 ^a,b^	0.010 ± 0.000 ^b–d^

Note: Values expressed as mean ± standard deviation. Values in a row followed by different letters are significantly different at *p* < 0.05 (Tukey’s HSD test). Enrichment levels (Low–Mid–High): 5–7–9% powdered WG; 3–5–7% encapsulated WG; 20–30–40% WG extract.

**Table 3:** The content and true retention (TRI) of minerals in cooked pasta enriched with WG.

Pasta Formulation	Enrichment Level	K		Ca		Mg		Fe		Zn	
		(mg/kg d.m.)	TRI (%)	(mg/kg d.m.)	TRI (%)	(mg/kg d.m.)	TRI (%)	(mg/kg d.m.)	TRI (%)	(mg/kg d.m.)	TRI (%)
Control	0	403.51 ± 0.05 ^a^	46.8 ± 0.3 ^f^	306.02 ± 15.82 ^a–c^	97.4 ± 0.8 ^b^	409.82 ± 4.17 ^b^	98.0 ± 0.6 ^d^	15.09 ± 0.08 ^a^	87.8 ± 1.2 ^a^	10.24 ± 0.42 ^a^	102.8 ± 1.6 ^c^
Powdered WG	Low	964.85 ± 7.98 ^e^	48.7 ± 1.0 ^f^	345.02 ± 7.96 ^d,e^	104.2 ± 2.2 ^e^	475.02 ± 4.40 ^f^	94.0 ± 2.6 ^c^	27.13 ± 0.79 ^d^	91.3 ± 6.3 ^a–c^	13.08 ± 0.11 ^b,c^	83.7 ± 1.9 ^a^
	Mid	1081.81 ± 6.09 ^f^	32.7 ± 0.2 ^c,d^	369.22 ± 4.90 ^e^	101.7 ± 0.7 ^c–e^	508.26 ± 1.18 ^h^	89.9 ± 0.8 ^b^	29.54 ± 0.49 ^e^	94.9 ± 2.7 ^a–c^	12.67 ± 0.27 ^b^	92.7 ± 2.8 ^b^
	High	1210.59 ± 19.48 ^g^	32.6 ± 0.4 ^c,d^	367.77 ± 0.14 ^e^	98.58 ± 0.4 ^b,c^	503.12 ± 3.42 ^g^	87.2 ± 0.3 ^b^	32.29 ± 0.36 ^f^	98.3 ± 1.0 ^c^	13.14 ± 0.41 ^b,c^	95.4 ± 4.4 ^b^
Encapsulated WG	Low	552.68 ± 4.56 ^b,c^	33.7 ± 0.7 ^d^	303.98 ± 21.14 ^a^	91.0 ± 0.6 ^a^	393.90 ± 3.78 ^a^	87.8 ± 1.1 ^b^	21.13 ± 0.27 ^b^	88.9 ± 1.4 ^a,b^	14.04 ± 0.22 ^c,d^	103.9 ± 0.8 ^c^
	Mid	576.56 ± 15.98 ^c^	24.5 ± 0.3 ^a^	328.13 ± 2.95 ^a,b^	96.2 ± 0.8 ^b^	401.94 ± 4.05 ^a,b^	83.4 ± 0.4 ^a^	23.97 ± 0.14 ^c^	87.8 ± 0.6 ^a^	14.28 ± 0.01 ^c,d^	92.6 ± 0.8 ^b^
	High	796.21 ± 7.88 ^d^	29.4 ± 0.3 ^b^	339.98 ± 6.20 ^a–d^	90.2 ± 0.9 ^a^	420.94 ± 9.63 ^c,d^	81.5 ± 1.1 ^a^	26.12 ± 0.11 ^d^	94.5 ± 3.7 ^a–c^	15.23 ± 0.12 ^d^	90.1 ± 0.9 ^a,b^
WG extract	Low	526.48 ± 20.75 ^b^	30.2 ± 0.0 ^b,c^	308.10 ± 3.58 ^a^	101.6 ± 0.4 ^c–e^	432.04 ± 2.48 ^d,e^	99.6 ± 1.6 ^d^	16.00 ± 0.52 ^a^	95.6 ± 1.2 ^b,c^	10.33 ± 0.07 ^a^	103.0 ± 1.1 ^c^
	Mid	572.47 ± 12.11 ^c^	39.9 ± 2.3 ^e^	320.78 ± 12.33 ^c,d^	103.4 ± 2.7 ^d,e^	440.29 ± 0.43 ^e^	100.7 ± 0.4 ^d^	16.21 ± 0.35 ^a^	93.4 ± 3.2 ^a–c^	10.74 ± 0.07 ^a^	106.4 ± 0.8 ^c^
	High	807.94 ± 1.20 ^d^	42.5 ± 0.5 ^e^	337.78 ± 2.22 ^c–e^	99.9 ± 0.2 ^b–d^	470.12 ± 0.29 ^f^	98.1 ± 0.5 ^d^	25.28 ± 0.81 ^d^	96.3 ± 0.1 ^b,c^	10.91 ± 0.49 ^a^	107.2 ± 0.0 ^c^

Note: Values expressed as mean ± standard deviation. Values followed by different letters are significantly different at *p* < 0.05 (Tukey’s test). Enrichment levels (Low–Mid–High): 5–7–9% powdered WG; 3–5–7% encapsulated WG; 20–30–40% WG extract.

**Table 4:** Cooking quality and textural properties of pasta enriched with various wild garlic (WG) preparations.

Pasta Formulation	Enrichment Level	Water Absorption (%)	Cooking Loss (%)	Swelling Index	OCT (min)	Hardness (N)	Adhesiveness (Ns)
Control	0	170.0 ± 25 ^b,c^	6.10 ± 0.14 ^a^	3.48 ± 0.30 ^b–d^	13	95.45 ± 4.05 ^b,c,d^	1.41 ± 0.21 ^a^
Powdered WG	Low	110.7 ± 18 ^a^	7.42 ± 0.12 ^b^	2.43 ± 0.24 ^a^	5	103.20 ± 6.21 ^c,d^	2.82 ± 0.64 ^b,c^
	Mid	111.0 ± 15 ^a^	6.17 ± 0.18 ^a^	2.29 ± 0.34 ^a^	7	125.50 ± 9.36 ^f^	3.35 ± 0.74 ^d^
	High	127.2 ± 16 ^a,b^	7.99 ± 0.16 ^c^	2.86 ± 0.20 ^a–c^	10	119.08 ± 9.25 ^f^	3.78 ± 0.80 ^d^
Encapsulated WG	Low	149.5 ± 19 ^a–c^	7.39 ± 0.22 ^b^	3.00 ± 0.22 ^a–d^	11	78.43 ± 4.28 ^a^	1.72 ± 0.36 ^a,b^
	Mid	134.2 ± 20 ^a,b^	9.68 ± 0.18 ^d^	2.86 ± 0.27 ^a–c^	12	81.66 ± 2.95 ^a,b^	1.61 ± 0.37 ^a^
	High	112.3 ± 14 ^a^	9.65 ± 0.11 ^d^	2.71 ± 0.31 ^a,b^	10	112.71 ± 5.63 ^d,e,f^	2.86 ± 0.41 ^b–d^
WG extract	Low	188.4 ± 15 ^c^	7.92 ± 0.15 ^c^	3.71 ± 0.35 ^d^	10	93.22 ± 6.74 ^b,c^	1.46 ± 0.23 ^a^
	Mid	178.3 ± 22 ^b,c^	6.22 ± 0.13 ^a^	3.57 ± 0.23 ^c,d^	10	100.60 ± 3.28 ^c,d^	1.96 ± 0.27 ^a–c^
	High	170.7 ± 17 ^b,c^	5.98 ± 0.19 ^a^	3.57 ± 0.27 ^c,d^	10	117.08 ± 49.59 ^e,f^	3.47 ± 3.78 ^d^

Note: Values expressed as mean ± standard deviation. Values followed by different letters are significantly different at *p* < 0.05 (Tukey’s test). OCT—optimum cooking time. Enrichment levels (Low–Mid–High): 5–7–9% powdered wild garlic; 3–5–7% encapsulated wild garlic; 20–30–40% extract of wild garlic.

**Table 5:** Colour properties of non-cooked and cooked pasta enriched with wild garlic (WG).

Samples	Pasta Formulation	Suppl. Level	L*	a*	b*	Colour Example
Non-cooked pasta	Control	0	72.46 ± 1.46 ^h^	0.86 ± 0.50 ^e^	31.56 ± 1.19 ^g^	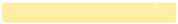
	Powdered WG	Low	45.64 ± 0.44 ^d^	−0.78 ± 0.09 ^c^	20.21 ± 0.77 ^b–d^	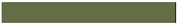
		Mid	41.07 ± 0.96 ^b,c^	−2.10 ± 0.10 ^a,b^	14.97 ± 0.36 ^a^	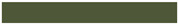
		High	41.40 ± 0.77 ^b,c^	−2.07 ± 0.16 ^a,b^	13.40 ± 0.74 ^a^	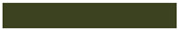
	Encapsulated WG	Low	51.21 ± 0.54 ^e^	9.13 ± 0.27 ^k^	25.64 ± 0.85 ^f^	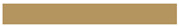
		Mid	52.62 ± 2.19 ^e^	8.17 ± 0.78 ^j,k^	23.26 ± 2.78 ^d–f^	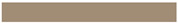
		High	54.73 ± 2.07 ^e^	8.58 ± 0.68 ^k^	27.25 ± 2.51 ^f^	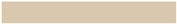
	WG extract	Low	66.78 ± 0.88 ^f,g^	3.90 ± 0.67 ^f^	34.79 ± 3.26 ^g,h^	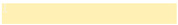
		Mid	63.84 ± 2.59 ^f^	5.05 ± 0.43 ^g^	33.95 ± 1.88 ^g,h^	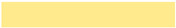
		High	66.30 ± 0.34 ^f,g^	4.38 ± 0.41 ^f,g^	35.41 ± 0.91 ^h^	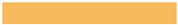
Cooked pasta	Control	0	72.59 ± 0.10 ^h^	−1.38 ± 0.28 ^b,c^	19.26 ± 0.15 ^b,c^	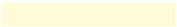
	Powdered WG	Low	40.39 ± 1.38 ^a–c^	−0.83 ± 0.11 ^c^	14.37 ± 1.37 ^a^	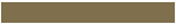
		Mid	37.64 ± 0.77 ^a^	−2.00 ± 0.16 ^a,b^	16.37 ± 0.54 ^a,b^	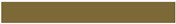
		High	38.34 ± 0.26 ^a,b^	−2.69 ± 0.16 ^a^	12.73 ± 0.39 ^a^	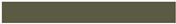
	Encapsulated WG	Low	52.21 ± 0.68 ^e^	7.07 ± 0.16 ^i,j^	21.29 ± 0.36 ^c–e^	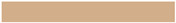
		Mid	43.16 ± 1.75 ^c,d^	6.63 ± 0.41 ^i^	16.91 ± 0.47 ^a,b^	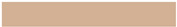
		High	38.26 ± 1.04 ^a,b^	6.86 ± 0.13 ^i^	14.70 ± 0.90 ^a^	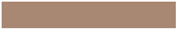
	WG extract	Low	71.96 ± 0.97 ^h^	−0.53 ± 0.22 ^c,d^	24.49 ± 0.32 ^e,f^	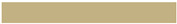
		Mid	67.52 ± 1.16 ^f,g^	1.02 ± 0.37 ^e^	25.70 ± 2.27 ^f^	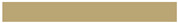
		High	67.84 ± 1.82 ^g^	0.37 ± 0.16 ^d,e^	25.65 ± 2.55 ^f^	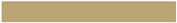

Note: Values expressed as mean ± standard deviation. Values followed by different letters are significantly different at *p* < 0.05 (Tukey’s test). Enrichment levels (Low–Mid–High): 5–7–9% powdered WG; 3–5–7% encapsulated WG; 20–30–40% WG extract.

## Data Availability

Data are contained within the article.
